# The Multifaceted Role of Aquaporin-9 in Health and Its Potential as a Clinical Biomarker

**DOI:** 10.3390/biom12070897

**Published:** 2022-06-27

**Authors:** Inês V. da Silva, Sabino Garra, Giuseppe Calamita, Graça Soveral

**Affiliations:** 1 Research Institute for Medicines (iMed.ULisboa), Faculty of Pharmacy, Universidade de Lisboa, 1649-003 Lisboa, Portugal; imsilva1@campus.ul.pt; 2 Department of Pharmaceutical Sciences and Medicines, Faculty of Pharmacy, Universidade de Lisboa, 1649-003 Lisboa, Portugal; 3 Department of Biosciences, Biotechnologies and Biopharmaceutics, University of Bari Aldo Moro, 70125 Bari, Italy; sabino.garra@uniba.it

**Keywords:** aquaporins, aquaporin-9, clinical biomarkers, membrane permeability

## Abstract

Aquaporins (AQPs) are transmembrane channels essential for water, energy, and redox homeostasis, with proven involvement in a variety of pathophysiological conditions such as edema, glaucoma, nephrogenic diabetes insipidus, oxidative stress, sepsis, cancer, and metabolic dysfunctions. The 13 AQPs present in humans are widely distributed in all body districts, drawing cell lineage-specific expression patterns closely related to cell native functions. Compelling evidence indicates that AQPs are proteins with great potential as biomarkers and targets for therapeutic intervention. Aquaporin-9 (AQP9) is the most expressed in the liver, with implications in general metabolic and redox balance due to its aquaglyceroporin and peroxiporin activities, facilitating glycerol and hydrogen peroxide (H_2_O_2_) diffusion across membranes. AQP9 is also expressed in other tissues, and their altered expression is described in several human diseases, such as liver injury, inflammation, cancer, infertility, and immune disorders. The present review compiles the current knowledge of AQP9 implication in diseases and highlights its potential as a new biomarker for diagnosis and prognosis in clinical medicine.

## 1. Introduction

**Figure 1:**
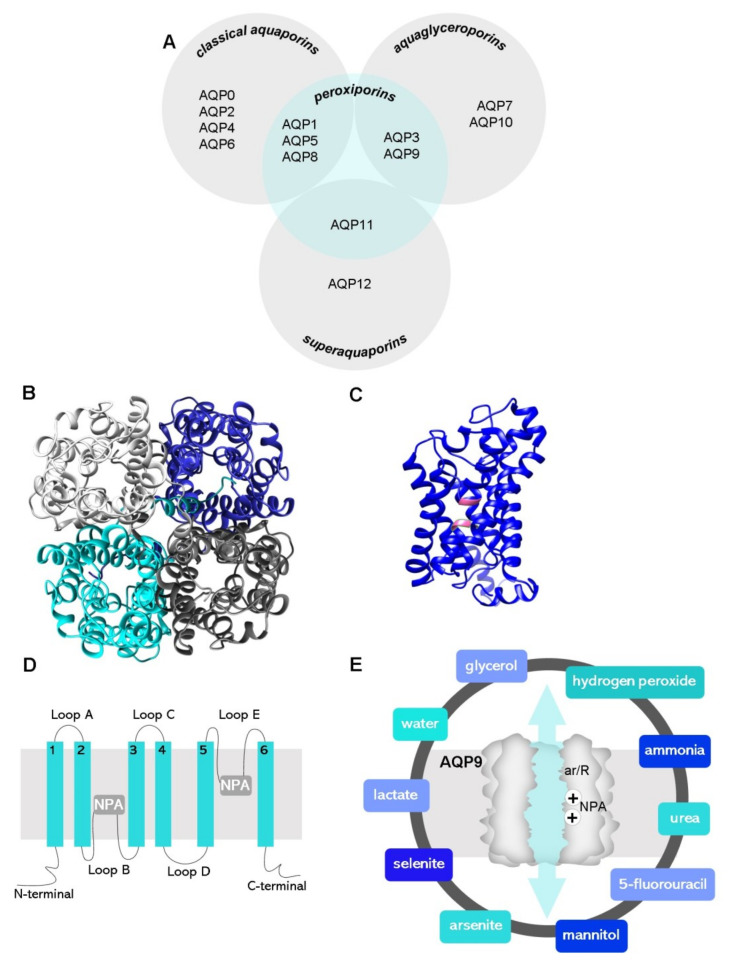
(**A**) General distribution of the 13 AQP paralogs according to their primary structure (grey circles) and selectivity. (**B**) Top view of the homotetrameric representation of the AQP9 glycerol channel and (**C**) side monomer view with the two NPA motifs (pink colored) based on a predictive structure generated in Phyre2 web portal. Final figures were generated using UCSF Chimera software. (**D**) Representation of AQP membrane topography, showing the monomer comprising six membrane-spanning α-helices (1–6) connected by five loops (A–E), the conserved asparagine–proline–alanine (NPA) motifs embed in the membrane. In the functional monomer, the hydrophilic loops B and E are bent back into the cavity formed by the two half-helixes. The two loops are spatially close to form the charge selective gate containing the two NPA motifs. (**E**) Graphical illustration of an AQP9 channel displaying the two selectivity filters: size and charge, ar/R and NPA, respectively, represented, and the molecules known to permeate the AQP9 channel.

Aquaporins (AQPs) are a family of transmembrane protein channels that facilitate the permeation of water and other small polar molecules (such as glycerol, urea, and hydrogen peroxide) driven by osmotic or solute gradients [1,2]. The 13 AQPs expressed in humans (AQP0–12) were roughly categorized into three main subgroups according to their selectivity and primary structure (Figure 1A) [3]: (1) classical or orthodox AQPs (AQP0, 1, 2, 4, 5, 6, 8), mainly water channels, although AQP6 and AQP8 also transport anions [4] and ammonia [5], respectively; (2) aquaglyceroporins (AQP3, 7, 9, 10), also allow movement of small non-charged molecules such as urea and glycerol; (3) superaquaporins or unorthodox aquaporins (AQP11 and 12), distinct from the other AQPs for their divergent evolutionary pathway, lower primary sequence homology, transport properties, and intracellular localization [6,7,8]. More recently, the reported permeability to hydrogen peroxide of several AQPs (AQP1, 3, 5, 8, 9, 11), gave rise to a fourth sub-group, partially overlapping with the other three sub-groups, named peroxiporins [9,10,11,12,13,14].

AQPs have been widely detected in all kinds of human tissues and cells, presenting cell-specific patterns according to their functions and modulation [15]. Every cell membrane may contain thousands of water channels, primarily allowing osmotic regulation [16]. Through analysis of *Aqp* knockout (KO) mice phenotype and the effects of their up- or down-regulation at the cellular level, other additional important roles have been uncovered. Classical AQPs are key regulators in several physiological functions such as water reabsorption and urine concentration in kidney [17] and brain water homeostasis [18], while aquaglyceroporins are crucial for glycerol metabolism, energy homeostasis [19,20,21], oxidative stress [22], and skin hydration [23,24]. Superaquaporins have been pointed as important cellular entities for organelle homeostasis, such as supporting endoplasmic reticulum function [14]. The extensive association of AQPs with a variety of important physiological roles and the described dysfunction in certain diseases, such as cancer, inflammation, and metabolic disorders [25,26], exposed these membrane channels as potential predictive and diagnostic biomarkers.

The aquaglyceroporin AQP9 is the main glycerol channel in hepatocytes involved in fat synthesis and gluconeogenesis, regulating body energy homeostasis [27]. Leukocytes are another major site of expression for this AQP. In recent years, AQP9 implication in a variety of disorders, including liver injury [28], inflammation [29], cancer [30,31,32,33,34,35], and altered immune response [36,37,38], revealed AQP9 as a promising drug target and a diagnostic/prognostic biomarker in clinical medicine.

## 2. Structure and Selectivity

Disclosure of AQPs’ three-dimensional structure enabled researchers to predict the protein channel fold and revealed the essential biophysical features for a specific permeation and selectivity. Human AQP1 was the first atomic structure resolved, enabling with the understanding of the structural features of other paralogs.

AQPs are constructed in membranes as homotetramers formed by four identical monomers (Figure 1B), each one behaving as an independent pore. Each monomer is constituted of 280-320 amino acids and has a molecular weight of around 28 kDa (Figure 1C). The AQP protein topology comprises six highly hydrophobic transmembrane domains (1–6) linked by five loops (A–E), two half-helixes containing highly conserved asparagine–proline–alanine (NPA) motifs representing the protein family signature, and cytosolic N-terminal and C-terminal sequences [39] (Figure 1D).

The selectivity of the channel is attained by two selectivity filters localized in the channel pore: (i) size-selective filters formed by aromatic/arginine residues (ar/R constriction site) near the extracellular vestibule that determine the size and hydrophilicity of molecules allowed to pass through (pore size of 2.8 Å for classical aquaporins and 3.4 Å for aquaglyceroporins) [40] and (ii) charge-selective filters formed by the two highly preserved NPA motifs that act as dipoles, avoiding ion permeation through the channel [41].

Recently, molecular dynamics simulations and other database-feed computational programs have been used as powerful tools to predict the unknown structure of several AQPs for drug design and molecular targeting. The homology model of human AQP9 generated, which is based on a predictive structure (Phyre2 web portal), is shown in Figure 1B,C for representation purposes.

Being primarily classified as an aquaglyceroporin, AQP9 has broad selectivity-facilitating transmembrane fluxes of water [42] and glycerol [43], as well as urea [42], hydrogen peroxide [13], lactate [44,45,46], mannitol [47], ammonia [48], arsenite [44,49], selenite [44], and 5-fluorouracil [50] (Figure 1E).

## 3. Tissue Distribution

**Figure 2:**
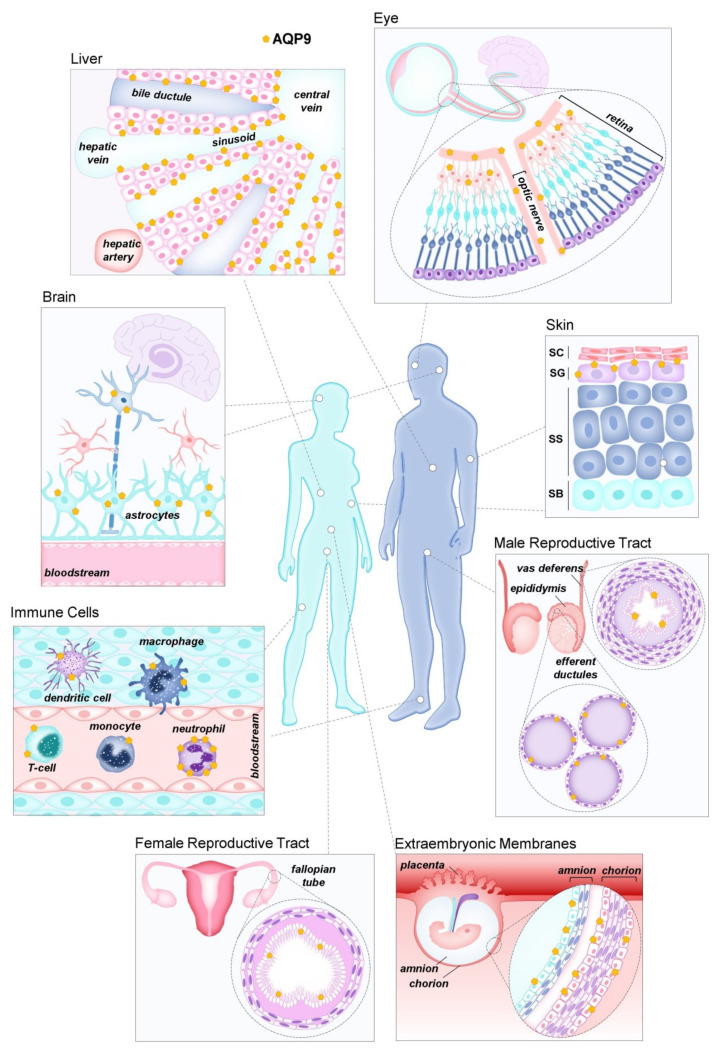
Aquaporin-9 (AQP9) distribution in human tissues. These tissues were reported to express AQP9, although several locations still need confirmation.

AQP9 was independently discovered in rats [51] and humans [42] where strong mRNA expression was found in liver and peripheral leukocytes in humans [42,52], as well as liver, Leydig cells, and immature spermatocytes in rats [51]. Since then, AQP9 immunoreactivity has been reported in many other tissues (Figure 2), including rat and human epididymis [53,54], various cell types of rodent and primate brain [53,55,56,57,58], mouse spinal cord [59], human astrocytes [60], human chorioamnion and placenta [61,62], human and mouse inner ear [63,64], human and mouse small intestine [65], human and rat prostate [66,67], human skeletal muscle [68], urothelium [69], porcine and rat oviduct [70], human fallopian tube [71], human adipose tissue [72], human retina [73,74], and human and mouse skin [75]. However, although some described differences between humans and rodents are likely due to species differences, the expression of AQP9 in some tissues is still debated. False positive identification of expression sites due to antibody cross-reactivity has been reported in a study where, in addition to using *Aqp9* knockout mice, a systematic analysis of the AQP9 expression within the Human Protein Atlas project tissue repository, including a large spectrum of normal and cancer tissues in addition to cell lines and primary cells, was run by shotgun RNA sequencing and protein immunohistochemistry [76]. Overall, the expression of AQP9 in humans appears to be more selective than in mice, with hepatocytes and neutrophils being by far the major expression sites in normal human tissues. Through the use of *Aqp9*-KO cells, AQP9 protein expression has been verified in hepatocytes [53,77,78,79], immune cells, including neutrophils [80,81], macrophages [82], CD8+ T-cells [83,84], stratum granulosum of the epidermis [75], principal cells of the epididymis, and vas deferens [77], bone marrow dendritic cells [37], and a limited population of neuronal cells [58,76,85].

## 4. Physiological Roles and Regulation

The reported physiological roles and modulation of human and rodent AQP9 in the tissues where its expression has been verified are summarized in Table 1.

In liver, AQP9 is mainly expressed in liver parenchyma, at the sinusoidal plasma membrane of hepatocytes [53]. In rat liver, AQP9 expression is stronger in the pericentrolobular region of the hepatic lobule than in the periportal area [77,86]. In rodents, AQP9 is the main pathway through which glycerol is imported from portal blood to hepatocytes during short-term fasting [78,79,87]. Once into the cells, glycerol is promptly converted into glycerol-3-phosphate (G3P) by glycerol kinase to be used as a substrate for gluconeogenesis. Hepatocyte AQP9 is also involved in lipid homeostasis as G3P is required for the synthesis of triacylglycerols (TAGs) [21]. AQP9 is also reported to be involved in rodent bile formation [88] and in the extrusion of catabolic urea [89]. In rodents, the expression of hepatocyte AQP9 is negatively regulated by insulin at the transcriptional level [90], likely explaining why hepatic AQP9 is increased in conditions of insulin resistance [91,92]. Important roles for AQP9 in glucose and lipid metabolism and energy homeostasis are also indicated by the phenotype expressed by *Aqp9* knockout mice where the lack of AQP9 leads to reduced liver glycerol permeability associated with increased levels of plasma glycerol and TAGs [77,89]. Estrogens have been seen to prevent the increase in hepatic AQP9 expression and glycerol uptake during starvation [89]. Mouse models of obesity and obese patients with type 2 diabetes showed reduced AQP9 levels in hepatocytes, with a significant decrease in liver glycerol permeability [93,94].

Hepatocyte AQP9 is also regulated by leptin at the gene transcriptional level [72,95]. However, the regulatory role of insulin and leptin on AQP9 appears to differ between rodents and humans [92]. Sex-specific dimorphism of hepatic AQP9 expression is found in both rodents and humans, and is consistent with the differences with which the two genders handle glycerol, glucose, and TAGs in energy metabolism [96].

In the immune system, AQP9 is expressed in neutrophils and a series of other cell types where it has been reported to play several roles. In neutrophils, AQP9 is required for the development of sensitization during murine cutaneous-acquired immune responses via regulation of cell function [80]. A function of AQP9 in F-actin polymerization influencing both morphologic and functional (i.e., cell migration) changes of neutrophils has also been suggested to explain its increased expression in patients with systemic inflammatory response syndrome compared to healthy subjects [81]. In line with previous research indicating functional involvement of AQP9 in murine bone marrow dendritic cell maturation in response to inflammatory stimulation [37] and in LPS-induced endotoxemic shock [29], a role in multisystemic neutrophil infiltration has been recently reported in a study using a murine model of polymicrobial infection. In these instances, AQP9 was required for the activation of NF-ĸB and the expression of the NLRP3 inflammasome, providing new and important clues on the signaling pathways through which AQP9 is involved in infiltration processes [97]. In macrophages, AQP9 was shown to be required in phagocytosis promoted by the *Pseudomonas aeruginosa* lasI/rhlI quorum sensing genes as its expression was strongly increased and the related protein was redistributed to the leading and trailing regions after macrophages were infected with the pathogen [82]. Involvement of AQP9 in macrophage M2 polarization has been recently shown in the progression of kidney renal clear cell carcinoma [84]. In CD8+ T cells, AQP9 was required for longevity and fast response to rechallenge [83], and to cell locomotion in reaching the tumor microenvironment [84].

Less defined is the role played by AQP9 in the other tissues where this aquaglyceroporin/peroxiporin is expressed. In the stratum granulosum of the epidermis, a skin layer where tight junctions act as a paracellular barrier against small molecules, AQP9 was suggested to represent a transcellular route for the movement of solutes of biological relevance, such as glycerol and urea, into and out of the skin, and be involved in the terminal differentiation of keratinocytes [23,24,25,75]. In the principal cells of the epididymis and vas deferens, AQP9 is thought to have implications in sperm concentration and storage, respectively [54]. The presence (cellular and subcellular localization) and role of AQP9 in the brain have been matter of discussions [58], with arguments of putative species differences and the very limited population of neurons in which this AQP seems to be expressed [85].

**Table 1:** Sites of expression, physiological roles and modulation of human and rodent AQP9.

Organ/Tissue (Species)	Cell Type	Suggested Physiological Functions	Modulation	References
Liver (h, m, r)	Hepatocytes	Glycerol uptake; lipid and glucose homeostasis; energy balance; primary bile secretion; extrusion of catabolic urea	Insulin (↓) *; estrogens (↓); leptin (↓)	[21,72,78,79,88,89,90,93,94,95]
Immune system (h, m)	Neutrophils	Development of sensitization during cutaneous-acquired immune responses (contact hypersensitivity)		[80]
		Cell migration		[81]
		Multisystemic tissue infiltration		[97]
		NF-KB pathway and NLRP3 activation		[97]
	Bone marrow dendritic cells	Cell maturation and inflammatory cytokine secretion		[37]
	Macrophages	Phagocytosis of bacteria		[82]
		Cell polarization and migration of M2 macrophages		[84]
	CD8^+^ T cells	Cell longevity		[83]
		Response to rechallenge		[83]
		Cell locomotion		[84]
Skin (h, m)	Upper keratinocytes of the stratum granulosum of epidermis	Glycerol and urea transcellular skin permeability		[23,24,25,75]
		Cell differentiation		[23,24,25,75]
Male reproductive tract (m, r)	Epididymal principal cells	Sperm concentration (?)		[54]
	Vas deferens principal cells	Sperm storage (?)		[54]
Female reproductive tract (h, r)	Fallopian tube epithelial cells	Water flow in oviductal lumen (?)	Estrogen and progesterone (?)	[71,98]
Brain (h, m)	Astrocytes (?) and catecholaminergic neurons (?)	Energy homeostasis (?)		[60,85]

h, human; m, mouse; r, rat; ↑/↓, increase/decrease in AQP9 expression. *, regulation in rodent liver (in humans, insulin was reported to upregulate AQP9 in hepatocytes through the phosphatidylinositol 3-kinase/Akt/mammalian target of rapamycin pathway [21]).

## 5. Potential Relevance of AQP9 as Clinical Biomarker

AQPs have a preponderant role in maintaining cell and tissue physiology, regulating membrane permeability, or influencing complex processes at a systemic level. Dysregulation of AQP9 is found in a variety of clinical conditions, such as cancer and inflammatory disorders. Table 2 summarizes the diseases in which AQP9 is thought to be involved and for which it could be used as a biomarker.

### 5.1. AQP9 in Liver Disease

Chronic liver injury (CLI), a common disease which is harmful to human health, generally manifests as hepatic steatosis and secondary chronic steatohepatitis, and are forms of diseases that can develop to liver fibrosis, cirrhosis and further, to hepatocellular carcinoma (HCC) if not treated in due time [28].

Various animal models of experimental CLI have been used to investigate the mechanisms of liver injury and the related involvement of AQP9, revealing this channel as an important biomarker of disease progression. Moreover, AQP9 overexpression in CLI makes this protein a target. *Aqp9* KO mouse models showed that silencing *Aqp9* alleviated hepatic lipotoxicity, protecting from downstream inflammation, oxidative stress, apoptosis, and pyroptosis [28].

Majority of the primary hepatic cancer burden pertains to HCC, with liver cancer representing the sixth most common oncologic condition and the second-leading cause of death from malignant neoplasm around the world [99]. AQP9 was shown to be consistently downregulated in HCC tissues and cells compared to normal liver tissues and hepatocyte cell lines, respectively [30,31,32,33,34,35]. Interestingly, AQP9-overexpressing human HCC SMMC-7721 cells displayed increased expression of E-cadherin and decreased expression of N-cadherin in vitro and in xenografted tumors that were positively correlated with decreased levels of phosphoinositide 3-kinase (PI3K) and p-Akt. In addition, AQP9 overexpression prevented cell invasion, proliferation, and migration in vitro and tumor growth in vivo, suggesting direct involvement of AQP9 in the settings of hepatocellular carcinoma development by influencing epithelial-to-mesenchymal transition [30,31,33,34]. Although AQP9 is consistently reported to be downregulated in HCC, the migration rate of HCC HepG2 cells was enhanced by the oleic acid-induced increased expression of AQP9, suggesting AQP9 as an important player in HCC metastasis [100]. AQP9 downregulation was also correlated with tumor extension, survival rate, and lymphatic and distal metastasis. Moreover, when overexpressing AQP9 in HCC cells, the levels of Wnt/β-catenin signaling and EMT-associated molecules were diminished while cell apoptosis was increased, suggesting the possible relevance of AQP9 as both a drug target and diagnostic molecule in HCC [31,33]. In this context, gene set enrichment analysis and LinkedOmics showed an important involvement of AQP9 in the most significant hallmark pathways. The microRNAs mir-23a-3p and mir-330-3p were correlated with AQP9 inhibition upon HCC. Moreover, AQP9 was shown to be critical in tumor immunity in liver cancer [32].

In a study focused on HCC patients without or with cirrhosis, AQP9 was found to be decreased in tumoral masses, but differential expression was described in non-tumorigenic liver (NTL) portions of the same donor. AQP9 remained membrane-localized with zonal distribution in the majority of NTL of non-cirrhotic patients while extensive AQP9 staining was observed in membranes. These data may represent valuable resources in future diagnostic strategies [35].

CD133+ liver cancer stem cells (LCSCs) show deficient AQP9 expression. Overexpressing AQP9 in LCSCs increases reactive oxygen species (ROS) accumulation promoting apoptosis and suppresses stemness. AQP9 is downregulated by insulin-like growth factor 2 (IGF2) which contributes to the maintenance of LCSC stemness [34]. Since LCSCs are critical in HCC due to their aggressive behavior, the early detection of decreased AQP9 in CD133+ LCSCs may prevent the development of HCC.

Controversially, another study reported that dibutyryladenosine 3′,5′-cyclic monophosphate (dbcAMP) decreased AQP9 expression in SMMC-7721 cells compared to normal HL-7702 (L02) liver cells, inhibiting the proliferation of SMMC-7721 cells in vitro [101].

In a rat model of obstructive extrahepatic cholestasis subjected to bile duct ligation (BDL), AQP9 was decreased in the basolateral membranes and increased intracellularly, a finding that was consistent with the observed considerable basolateral membrane reduction in water and glycerol permeability of cholestatic hepatocytes [88]. The altered expression of liver AQP9 induced by the cholestasis did not depend on insulin, a hormone downregulating AQP9 at the transcriptional level, since insulinemia in BDL rats was unchanged. As also suggested for AQP8, another AQP reduced in BDL rats [102], the defective expression of AQP9 was hypothesized to contribute to primary bile secretory dysfunction in cholestatic liver [88].

Hepatic steatosis is the hallmark of non-alcoholic fatty liver disease (NAFLD). Starved obese leptin-deficient (ob/ob) mice, a model of NAFLD, displayed deficient AQP9 expression and function and increased levels of plasma glycerol compared to lean mice, suggesting the implication of AQP9 in liver steatosis. The same reduction in AQP9 expression was observed in human liver biopsies from morbid obese patients undergoing bariatric surgery [94], and has been pointed as a possible defensive mechanism to counteract further fat infiltration in liver parenchyma [93].

In an oleic acid-induced NAFLD LO2 cell model, AQP9 overexpression aggravates the degree of steatosis, while its silencing alleviates these effects [103]. A similar model developed with HepG2 showed concordant results [104]. Oleic acid treatment boosts the p38 phosphorylation, while the inhibition of p38 prevents AQP9 upregulation, suggesting AQP9 as an important player in oleic acid-induced hepatic steatosis in HepG2 cells via p38 signaling [104].

Steatosis, the hepatic manifestation of metabolic syndrome and insulin resistance, may evolve towards non-alcoholic steatohepatitis (NASH), cirrhosis, and hepatocellular carcinoma. Autophagy and oxidative stress are intrinsic to hepatic lipid accumulation and NAFLD progression. In an oleate/palmitate-induced rat hepatoma FaO cell model of NAFLD progression, hepatic lipid homeostasis is perturbed by TNFα treatment. However, silybin, a lipid-lowering nutraceutical, ameliorates fatty acids profile of lipid droplets, stimulates mitochondrial oxidation, upregulates miR-122, a microRNA of pivotal relevance in hepatic fat metabolism, and restores the levels of AQP9 and glycerol/hydrogen peroxide permeability while reducing the activation of the oxidative stress-dependent transcription factor NF-κB and autophagy turnover. These data revealed signaling pathways that relate AQP9 to hepatocyte protection, by their involved with TAGs metabolism, fat-induced autophagy, and AQP9-mediated glycerol transport in hepatocytes [105].

Ornithine transcarbamylase deficiency (OTCD), a urea cycle disorder, is characterized by insufficient AQP9 expression. The forced expression of AQP9 in patient-derived human-induced pluripotent stem cells differentiated into hepatocytes (hiPSCs-Heps), an in vitro model of OTCD, was shown to be effective in normalizing ureagenesis [106].

Therefore, AQP9 stands as an important biomarker for diagnosis and prognosis in liver diseases, especially HCC.

### 5.2. AQP9 in Inflammation and Immune Disease

AQP9 is one of the most expressed AQPs in immune cells—lymphocytes, neutrophils, and monocytes—and is closely related to the ability of these cell types to promptly respond after sensing danger signals by promoting rapid morphological modifications.

In human leukocytes, AQP9 increases after intravenous or in vitro LPS stimulation [38,107]. The AQP9 expression level also augmented in activated polymorphonuclear leukocytes in subjects with systemic inflammatory response syndrome (SIRS) [81] and infective endocarditis [108]. In dendritic cells (DCs), AQP9 was significantly augmented by LPS stimulation. In *Aqp9* KO mice with induced colitis, AQP9 gating did not completely protect from colitis-related inflammation but reduced the inflammatory response of DCs [37]. Human neutrophils and primary blood-derived macrophages showed abundant AQP9, and upregulation at both transcript and protein levels was observed after stimulation with LPS [36]. In addition, patients with SIRS showed increased AQP9 levels in neutrophils compared to healthy control subjects [81].

AQP9 was also suggested to be one of the most relevant AQPs in the immune system by regulating the migration of different immune cell types [109]. AQP9 regulates neutrophil cell migration and impacts sepsis survival [109]. In leukocytes, AQP9 was found at the cell edges, likely to facilitate motility, lamellipodium extension and stabilization, and cell volume changes, enabling these cells to migrate toward chemoattractants [110]. *Pseudomonas aeruginosa*-induced upregulation of AQP9 in human macrophages triggers changes in macrophage size and morphology, an outcome of critical importance in cell motility, migration, and phagocytosis [82]. In virus-activated memory CD8+ T cells, but not naive cells, IL-7-induced AQP9 upregulation impacts long-term cell longevity and homeostasis [83]. In a murine model of skin-allergic contact dermatitis using *Aqp9* KO mice, recruitment of neutrophils was attenuated, and locomotive ability was decreased. Furthermore, neutrophil deficiency in *Aqp9* KO mice induced a decrease in IL-17A production by draining lymph node cells, resulting in low T cell activation [80].

In human acute promyelocytic leukemia (APL) cell lines, NB4 and HT93A, and primary APL cells, the expression level of AQP9, rather than other biomarkers, correlated with sensitivity to arsenic trioxide in both APL cell lines and primary cells. These observations suggest the AQP9 expression status of APL patients as being a potent predictive marker for successful arsenic trioxide-based treatment, since AQP9 represents arsenite transport activity [111].

Clear cell renal cell carcinoma (CCRCC) has an important immune reactivity component associated with its carcinogenesis. CCRCC biopsies displayed increased transcriptional and proteomic AQP9 expressions, with transcriptional levels associated with aggressive progression, poor survival, and immune infiltration [112]. This leads to the hypothesis that AQP9 is an oncogene and a promising prognostic marker in CCRCC.

Sepsis is a severe pathological condition caused by systemic infection and is a major health concern as it is the primary cause of death from infection. It is the leading cause of mortality worldwide and there are no specific effective treatments for sepsis. *Aqp9* KO mice submitted to LPS-induced endotoxic shock after LPS challenge survived longer than controls, and 25% of *Aqp9* KO mice completely recovered. In addition, the AQP9-depleted mice displayed reduced inflammatory NO and O_2_^-^ production and iNOS and COX-2 levels, achieved by decreased NF-κB p65 activation. These data suggest that AQP9 plays an important role in the early acute phase of LPS-induced endotoxic shock involving NF-κB signaling [29]. AQP9 has been recently demonstrated to have an important pathophysiological role in sepsis, since RG100204, a novel, potent and selective AQP9 inhibitor, reduced septic cardiomyopathy and multiple organ failure in a cecal ligation and puncture (CLP) induced murine model of polymicrobial infection [97]. RG100204 was found to reduce the activation of NF-ĸB as well as the expression of the NLRP3 inflammasome in the heart and kidney. Moreover, CLP mice showed a significant increase in the activity of myeloperoxidase, an enzyme stored in granules of neutrophils that are released upon neutrophil activation during inflammation, in the lungs, which was effectively attenuated by RG100204, hence indicating the protective effects of RG100204 on the lungs. Together, these results indicated that AQP9 may be a novel drug target in polymicrobial sepsis and, at the same time, a valuable biomarker of this worrisome condition.

### 5.3. AQP9 in Infertility Disorders and Pregnancy Complications

#### 5.3.1. Female

Despite the still doubtful applicability of AQP9 as a clinical biomarker, few studies have debated its altered expression in both female and male infertility and pregnancy complications (see [113] for review). For example, in polycystic ovary syndrome (PCOS), decreased AQP9 expression in the granulosa cells was associated with hyperandrogenism [114,115], influencing the maturation of PCOS follicles [115]. In gestational diabetes mellitus, placentas from cesarean delivery at term showed higher expression of AQP9 compared to normal placentas [116]. In pre-eclampsia, serum AQP9 was increased in the early onset of the disease compared to healthy women [47,117]. The analysis of the area under the ROC curve (AUC) demonstrates that the serum AQP9 value can be considered a stable biomarker in pre-eclampsia early-onset diagnosis by depicting the inflammatory state of the patient [117]. In tubal ectopic pregnancy patients, the fallopian tubes showed lower expression levels of AQP9 than in healthy subjects [71]. In addition, in polyhydramnios, AQP9 expression was associated with excessive accumulation of amniotic fluid and was found to be increased in amnion and chorion and decreased in the placenta of women of term pregnancies [118].

#### 5.3.2. Male

Sertoli cells guarantee an adequate environment inside the seminiferous tubule for spermatogenesis to occur. High 17β-estradiol is known to interfere with the process. In mouse Sertoli cells, AQP9 expression and glycerol transport was impaired by estradiol treatment. Estradiol seems to influence the physiology of Sertoli cells and spermatogenesis through its influence over AQP9; thus, alterations in estradiol levels and AQP9 function may have implications in male infertility [119].

The exposure to endocrine disruptors during neonatal life was associated with increased incidence of testicular dysgenesis syndrome, a male reproduction-related condition characterized by hypospadias, cryptorchidism, poor semen quality, and testicular cancer. The neonatal exposure to diethylstilbestrol, a nonsteroidal estrogen, induces the upregulation of AQP9 in epididymal efferent ducts of male Spraque–Dawley rats [120,121]. Furthermore, rats treated with nonsteroidal antiandrogen flutamide and castrated adult rats showed lower levels of epididymal AQP9 compared to controls. Testosterone treatment promotes the recovery of AQP9 expression up to control levels [121,122], ensuring sperm maturation and storage.

The cystic fibrosis transmembrane conductance regulator (CFTR) is known to interact with [123] and control AQP9 [124], the most expressed AQP throughout the rat male reproductive tract. Impaired function of CFTR and AQP9 alter seminiferous tubular secretion and the formation of epididymal fluid. These findings open new insights on diagnosis and therapeutic targets to identify and reduce infertility in men with cystic fibrosis [123].

AQP9 activity as a lactate transporter makes this channel essential in primary spermatocytes and of maturing haploid germ cells since it is a key metabolite in the process. Human varicocele testis biopsies showed AQP9 downregulation, leading to lactate deprivation with subsequent hypospermatogenesis [125].

### 5.4. Potential Implication of AQP9 in Neuronal Disorders

Although the involvement of AQP9 in the nervous system has been scarcely addressed, a few studies have proposed its altered expression in human neuronal disorders and tumors. For instance, AQP9 overexpression was reported in human astrocytic tumors [60], and resulted in changes in astrocyte morphology, as previously described in astrogliosis processes after injury [126]. In human glioblastoma, AQP9 was strongly expressed in most glioma cell surfaces throughout the tumor [127], suggesting that AQP9 may represent a potential biomarker for this clinical condition.

**Table 2:** AQP9 involvement in diseases where it has potential to become a clinical biomarker.

Tissue	Cell	Species	Disorder/Pathology	Effect on AQP9 Expression/Function	References
Liver	Hepatocytes	h	Hepatocellular carcinoma (HCC)	↓	[30,35]
Liver	SMMC- 7721 Cells	h	Hepatocellular carcinoma (HCC)	↓	[30,33]
Liver	HLE, Huh-7, HepG2, SMMC-7721 Cells	h	Hepatocellular carcinoma (HCC)	↓	[31]
Liver	Huh7, SNU182, Li-7, Hep3B Cells	h	Hepatocellular carcinoma (HCC)	↓	[32]
Liver	Hepatocytes	r	Hepatocellular carcinoma (HCC)	↓	[31]
Liver	Hepatocytes	h	Non-alcoholic fatty liver disease (NAFLD) and steatohepatitis (NASH) (in obese T2D patients)	↓	[94]
Liver	Hepatocytes	m	NAFLD	↓ (in leptin-deficient animals)	[93]
Liver	HepG2	h	Steatosis	Increased (with oleic acid treatment)	[104]
Liver	LO2 Cells	h	Steatosis	Increased (with oleic acid treatment)	[103]
Liver	Hepatocytes	r	Obstructive extrahepatic cholestasis	↓	[88]
Kidney	Renal Clear Cell	h	Renal clear cell carcinoma	↑	[112]
Kidney	M2 Macrophages	h	Renal clear cell carcinoma	↑	[84]
Eye	RGC-5 Cells, Primary Retinal Ganglion Cells, Retina	h	Neurological disorders	↑	[128]
Eye	Retina	r	Glaucoma	↑	[73]
Brain	Astrocytes	m	Stroke	↑ before, ↓ after (*in vitro* with thrombin treatment)	[126]
Brain	Astrocytes	m	Focal transient ischemia	↑	[55]
Brain	Astrocytes	r	Glioblastoma	↑	[127]
Brain	Astrocytes	h	Astrocytic tumor	↑	[60]
Hippocampus and cerebral cortex	Astrocytes	h, m	Alzheimer disease	↓ (*in vitro* with amyloid-beta protein treatment)	[129]
Blood	Leucocytes	h	Acute promyelocytic leukemia	↑	[130]
Blood	Leucocytes	h	SIRS	↑	[81]
Blood	Dendritic cells	m	Inflammation activation	↑	[37]
Blood	Macrophages	h	Infection	↑	[82]
Blood	Leucocytes	h	Inflammation activation	↑	[38]
Blood	Monocytes	h	Inflammation activation	↑	[36]
Fetal membrane and placenta	Amnion epithelia, chorion cytotrophoblasts	h	Polyhydramnios	↑	[118]
Fetal membrane and placenta	Placental trophoblast	h	Polyhydramnios	↓	[118]
Placenta	Syncytiotrophoblast	h	Preeclampsia	↑ expression ↓ function	[47,117]
Placenta	Syncytiotrophoblast	h	Gestational diabetes	↑	[116]
Female reproductive system	Fallopian tube epithelial cells	h	Tubal ectopic pregnancy	↓	[71]
Female reproductive system	Granulosa cells	h	Polycystic ovary syndrome (PCOS)	↓	[114,115]
Male reproductive system	Epididymis	r	Infertility	↓ (by decreased function of cystic fibrosis transmembrane conductance regulator, CFTR)	[124]
Male reproductive system	Sertoli cells	m	Infertility	↓ (with increased high 17β-estradiol, E2)	[119]
Male reproductive system	Sertoli and germ cells	h	Varicocele, infertility	↓	[125]
Lung	Non-small cell	h	Non-small cell lung cancer	↑	[131]

h, human; m, mouse; r, rat; ↑/↓, increase/decrease in AQP9 expression.

## 6. Final Remarks

Growing evidence suggests that the assessment of AQP9 expression has great potential in providing valuable information, in addition to other biomarkers that, as yet are unavailable, for several disorders. The distribution of AQP9 in mammalian tissues and its implication in a broad range of pathophysiological conditions make this protein a promising biomarker in medical diagnosis. However, several findings and correlations herein require validation at the bench level and, most importantly, in retrospective and prospective clinical studies. Aside from clinical validation, evaluating AQP9 expression will require investment in robust and cost-affordable assays to implement its usefulness as a disease biomarker. Nevertheless, detection of AQP9 alone or combined with other biomarkers improving their accuracy, particularly for early diagnosis, may contribute to an increased survival rate in diseases such as cancer and sepsis. Nevertheless, detection of AQP9 alone or combined with other biomarkers improving their accuracy, particularly for early diagnosis, may contribute to an increased survival rate in diseases such as cancer and sepsis.

## Data Availability

Not applicable.
